# Maintenance bevacizumab beyond first-line paclitaxel plus bevacizumab in patients with Her2-negative hormone receptor-positive metastatic breast cancer: efficacy in combination with hormonal therapy

**DOI:** 10.1186/1471-2407-12-482

**Published:** 2012-10-19

**Authors:** Alessandra Fabi, Michelangelo Russillo, Gianluigi Ferretti, Giulio Metro, Cecilia Nisticò, Paola Papaldo, Ferdinando De Vita, Giuliana D’Auria, Antonello Vidiri, Diana Giannarelli, Francesco Cognetti

**Affiliations:** 1 Division of Medical Oncology A, Regina Elena National Cancer Institute, Via Elio Chianesi 53, Rome, Italy; 2 Division of Medical Oncology, Second University of Naples School of Medicine, II Policlinico, Naples, Italy; 3 Division of Medical Oncology, Belcolle Hospital, Viterbo, Italy; 4 Diagnostic Imaging Department, Regina Elena National Cancer Institute, Rome, Italy; 5 Service of Biostatistics, Regina Elena National Cancer Institute, Rome, Italy

**Keywords:** Maintenance Bevacizumab, Antiangiogenic agents, HER2 negative metastatic breast cancer

## Abstract

**Background:**

Data on efficacy of bevacizumab (B) beyond first-line taxane -including regimen (BT) as first-line treatment are lacking. Although preclinical results that anti-angiogenic agents combined with hormonal therapy (HT) could be active, no clinical data exist about combination of maintenance Bevacizumab (mBev) with HT.

**Methods:**

Thirty-five patients who experienced a response after first-line BT, were given mBev at the dose of 15 mg/kg every 3 weeks. Among 30 pts with hormonal receptor-positive metastatic breast cancer (MBC), 20 (66.6%) received HT with mBev (mHTBev). Objective of the study was the outcome and safety of mBev and in two groups of patients receiving HT or not.

**Results:**

Complete response and partial response was achieved/maintained in 4 (11.4%) and 13 (37.1%) patients, respectively (overall response rate: 48.5%). Clinical benefit was obtained on 23 patients (65.7%). Median of mBev PFS and clinical benefit were 6.8 months (95% CI: 0.8-12.7) and 17.1 months (95% CI :12.2-21.9), respectively. Median PFS of patients who received mHTBev was longer than mBev without HT (13 months and 4.1 months, respectively, p = 0.05). The most common severe toxicities were proteinuria (11.4%) and hypertension (8.5%). No additional toxicity was observed with HTBev.

**Conclusion:**

Maintenance bevacizumab with or without anti-hormonal therapy in patients with hormone receptor positive breast cancer is tolerable and associated with long-term clinical outcome; these results encourage the strategy of prolonging bevacizumab until progression in combination with anti-hormonal agents.

## Background

Angiogenesis is one of the key mechanisms of tumor growth and survival and is necessary for cancer rising, invasion and metastatization. The mechanism of angiogenisis is regulated by some pro-angiogenic factors such as the vascular endothelial growth factor (VEGF) [1,2]. The VEGF, over expressed in many tumors and associated with poor prognosis, is an attractive target for the development of biological therapy [3,4]. Bevacizumab (Avastin®), a recombinant humanized monoclonal antibody directed against VEGF, is currently approved for the treatment of many solid tumors and it represents a valid option for treatment of HER2-negative metastatic breast cancer (mBC) patients [5,6]. In advanced disease, the efficacy of first-line bevacizumab including chemotherapy has been proven in three randomized clinical trials [7-9] and its activity has been recently shown as second-line option [10]. Overall, the addition of bevacizumab to chemotherapy resulted in a longer progression-free-survival (PFS) and higher objective response rates (RRs), without any improvement in overall survival (OS). Thus, every research effort should be to improve the efficacy of every first-line bevacizumab-including regimen.

An attractive option could be to continue the anti-angiogenic agent as maintenance therapy in patients who are responder to first-line bevacizumab-based chemotherapy. This approach has an interesting preclinical rationale derived from studies that have suggested the power to increase tumor growth following withdrawal of VEGF inhibitors. In fact, although the VEGF inhibitors can destroy as much as 80% of tumor vasculature, tumor vessels may rapidly re-grow after cessation of treatment with these inhibitors [11,12]. Recently, Mancuso et al. [13] have also shown that when tumors in transgenic mouse were inhibited by VEGF tyrosine kinase receptor the same tumors were completely re-vascularized within the first week after stopping treatment, indicating that surviving pericytes and the empty sleeves of vascular basement membrane contributed to the rapid restoration of the tumor vasculature.

These preclinical data and the emerging results in advanced colo-rectal cancer [14,15], findings suggesting the clinical benefit of bevacizumab beyond first-line therapy due to permanent suppression of VEGF, could be an assumption for the clinical use of the biological agent as maintenance treatment also in mBC patients.

Preclinical models suggested that addition of anti-vascular endothelial growth factor therapy could improve the efficacy of anti-estrogens in hormone-sensitive breast cancer [16]. Recently a phase II trial in advanced breast cancer evaluated the feasibility and efficacy of bevacizumab added to either anastrozole or fulvestrant in postmenopausal hormonal receptor-positive patients already resistant to the adjuvant aromatase inhibitor. Both regimens showed a good response rate and encouraging progression free survival (ORR 28%, median PFS 18 months) without severe toxicity registered in both regimens [17].

In this study, we consecutively evaluated the safety and activity of maintenance therapy with bevacizumab alone or combined with endocrine therapy (HT), beyond response or disease stabilization by first-line combined chemotherapy (bevacizumab plus paclitaxel) in HER2-negative mBC patients.

## Methods

### Study design

The primary objectives of this prospective and observational study were: 1) to evaluate the activity of two consecutive groups of women receiving bevacizumab as maintenance (mBev) combined with hormonal therapy or not; 2) to assess the activity mBev in MBC patients responding to first-line paclitaxel-bevacizumab (BT).

Secondary objectives were: 1) to assess the safety profile of mBev; 2) to assess mBev progression free survival (PFS), clinical benefit duration and overall survival (mBev OS) of these MBC women.

### Patient population

All data of this multicenter study were collected at the Regina Elena National Cancer Institute in Rome. All patients provided written informed consent before undergoing any study-specific procedure. The study was approved by the Local Ethic Committee (IFO, Regina Elena Cancer Institute).

### Patients eligibility

HER2-negative mBC patients were included and prospectively followed. Patients were considered eligible if they had histological diagnosis of breast cancer, had metastatic disease, and had been never treated by chemotherapy and/or bevacizumab for advanced disease.

Patients might have a Performance Status (ECOG) ≤ 2 and a life expectancy ≥ 3 months. Patients with previously controlled neoplastic disease diagnosed over 5 years before were allowed to enter the study. They were neither pregnant nor nursing. Patients with symptomatic central nervous system metastases were excluded. They were required to have a left ventricular ejection fraction (LVEF) of 50% or greater, absolute neutrophil count more than 1500 x ll, platelet count more than 100.000 x ll, bilirubin less than 2 mg/dl, AST/ALT less than 3 times the upper normal limit (UNL). Other concurrent antineoplastic therapy was not admitted and patients might have concluded any radiotherapy or hormonal therapy at least 1 month before treatment. Patients could receive concomitant bisphosphonate treatment.

Patients were given bevacizumab 10 mg/kg iv on day 1 and 15 plus Paclitaxel 80 mg/mq iv on day 1, 8, 15, every 28 days as first-line therapy. Cycles were continued at discretion of investigator, but not interrupted before 6 cycles in case of response or stable disease. Assessment was performed every three courses. In case of response and/or stable disease, after 3 weeks of the last BT cycle, patients were given mBev therapy at the dose of 15 mg/kg every 21 days with or without hormonal agents, based on the expression of hormonal receptors. Maintenance bevacizumab (associated to hormonal treatment in hormonal receptors positive tumor) was used until disease progression, excluding patient’s withdrawal, unacceptable toxicity, deteriorated clinical conditions or patient’s refusal. Exclusion criteria for the use of maintenance hormonal therapy were as follows: patients in premenopausal status who had previously received tamoxifen as adjuvant treatment experiencing disease in course of adjuvant hormonal therapy; previous intolerance or onset of severe side effects to aromatase inhibitors; contraindications to receive tamoxifen in premenopausal patients (history of thromboembolism, intolerance, endometrial disease;); patients’ refusal.

### Study assessment

Evaluation of response was performed by Computed Tomography scan. The response to BT, the response to mBev, the mBev PFS (from the start of mBev to the first tumor relapse), the duration of clinical benefit (from the start of BT to the first relapse in course of mBev), the overall survival (OS, from the start of BT to death) were evaluated. RECIST criteria were used for response evaluation [18].

Tumor response and survival status were followed every 12 weeks both during BT treatment and mBev therapy. Complete response (CR) was defined as disappearance of all target lesions; partial response (PR) was defined as ≥ 30% decrease in the sum of the largest diameter of target lesions compared with the baseline; progressive disease (PD) was defined as ≥ 20% increase in the sum of the largest diameter of target lesions compared with the sum of largest diameter recorded before treatment start. Stable disease (SD) was defined as neither sufficient shrinkage to qualify for complete or partial response nor sufficient increase to qualify as progressive disease.

Information including demographics, medical history, breast cancer history, and tumor biology was collected. During treatment, all adverse events related to both BT and mBev were recorded on the electronic case report form. Data were collected for up to 12 months after the last bevacizumab infusion for the following specific adverse events previously reported in trials of bevacizumab: hypertension; proteinuria; arterial and venous thromboembolic events; congestive heart failure; central nervous system bleeding; other hemorrhages; wound-healing complications; gastrointestinal perforations and fistulae. Additional information on haematological toxicity was collected using incidence of National Cancer Institute Common Toxicity Criteria for Adverse Events (NCI CTC AE, version 3.0) [19].

### Statistical analysis

All patients enrolled were considered in the intention-to-treat population (ITT). This population was evaluated both for the efficacy and safety analysis. Rate and median times are reported with their 95% confidence interval to better interpret data.

The time to event analysis was performed according the Kaplan-Meier method and differences among curves were evaluated by the log-rank test. SPSS software version 17.0 was used for statistical analyses (SPSS, Inc. Chicago, Ill.)

## Results

### Patients characteristics

From July 2007 through May 2010, thirty-five patients entered the study. Their characteristics are listed in Table 1. The median age was 50 years (32–72) and 29 (82.9) patients had postmenopausal status. Most of women (85.7%) had tumors expressing hormonal receptors, twenty-two (62.8%) patients had previously received adjuvant chemotherapy, 9 (25.7%) of them were administered regimens including a taxane; 23 (65.7%) patients had visceral involvement.

**Table 1:** Characteristics of 35 HER2 negative mBC patients treated with Bevacizumab plus Paclitaxel followed by maintenance Bevacizumab

**Total number of patients (*n*** **= 35)**	**Frequency (%)**
Median age (range)	50 (32–72)
Menopausal Status	
Pre	6 (17.1)
Post	29 (82.9)
Hormonal Receptor Status	
Negative	5 (14.3)
Positive	30 (85.7)
Triple negative	5 (14.3)
Adjuvant chemotherapy	
- None	13 (37.1)
- including anthracyclines	9 (25.7)
- including antracyclines + taxanes	9 (25.7)
- Other regimens	4 (11.4)
Adjuvant hormonal therapy	22 (62.8)
Visceral metastatic sites	23 (65.7)
Median Disease Free Survival, months (range)	30 (0–362)
Median Cycles of Bevacizumab + Paclitaxel (range)	8 (6–12)
Median cycles of three-weekly mBev (range)	7 (1 – 28)
Median PS at the start of mBev (range)	0 (0–1)

PS, Performance Status (ECOG); Triple negative, estrogen receptor, progesteron receptor, HER2 negative; mBev, mainteinance Bevacizumab.

All patients received the BT combination, for a median of 8 cycles (6–12). In all cases the treatment was followed by mBev. The median of mBev three-weekly cycles given was 7 (1–28).

Among 30 patients with hormonal receptor-positive mBC, mBEV combined with hormonal therapy (HT) was given to 20 (66.6%) post-menopausal patients (19 of them received an aromatase inhibitor and 1 tamoxifen); one of the exclusion criteria for using maintenance HT was present in the remained 10 hormonal receptor positive patients.

### Efficacy of BT

All patients were evaluable for efficacy. Complete response and partial response was achieved in 4 (11.4%) and 18 (51.4%) patients, respectively, for an overall response rate of 62.8% (95%CI: 46.8-78.8); stable disease was shown in 13 (37.2%) patients.

### Efficacy of maintenance Bevacizumab

All patients were evaluable for efficacy. CR and PR was achieved/maintained in 4 (11.4%) and 13 (37.1%) patients, respectively, for an overall response rate of 48.5%; ten (28.6%) women experienced SD. Clinical benefit was obtained on 23 patients (65.7%). Eight (22.9%) patients progressed during mBev therapy.

Table 2 shows the activity of mBev according to response to BT. All CRs achieved in course of BT treatment were maintained during mBev; among 18 patients achieving PR by BT, 12 (66.6%) maintained responses during mBev, while 6 (33.4%) experienced PD. Among 13 patients showing SD while on BT, PR, SD and PD in course of mBev were obtained in 1 (7.7%), 10 (76.9%) and 2 (15.4%) patients, respectively.

**Table 2:** Response to maintenance Bevacizumab (mBev) according to activity of Bevacizumab plus Paclitaxel (BT)

	**Response mBev**			
**Activity BT**	**CR**	**PR**	**SD**	**PD**
***n***	***n*(%)**	***n*(%)**	***n*(%)**	***n*(%)**
4 CR	4 (100)			
18 PR		12 (66.7)		6 (33.3)
13 SD		1 (7.7)	10 (76.9)	2 (15.4)

mBev, maintenance Bevacizumab; CR, complete response; PR, partial response; SD, stable disease; PD, progression disease.

The median mBev PFS was 6.8 months (95%CI: 0.8-12.7). The median mBev PFS was 10.2 months among patients who received a taxane as adjuvant treatment compared with 6.6 months among patients who did not. At a median follow up of 17.5 months (10–49), median duration of clinical benefit was 17.1 months (95%CI :12.2-21.9). The median OS has not been reached yet.

### Efficacy of maintenance Bevacizumab according to hormonal therapy

Among patients given mBev plus anti-hormonal therapy (HTBev), 12 (60%) patients responded to treatment including 3 CR (15%) and 9 PR (45%). Stable disease was achieved in 6 (30%) patients, while 2 (10%) experienced PD. Among patients receiving mBev alone, 5 (33%) achieved response, including 1 CR (6.6%) and 4 PR (26.7%); stable and progression disease were seen in 4 (26.7%) and 6 (40%) patients, respectively.

**Figure 1:**
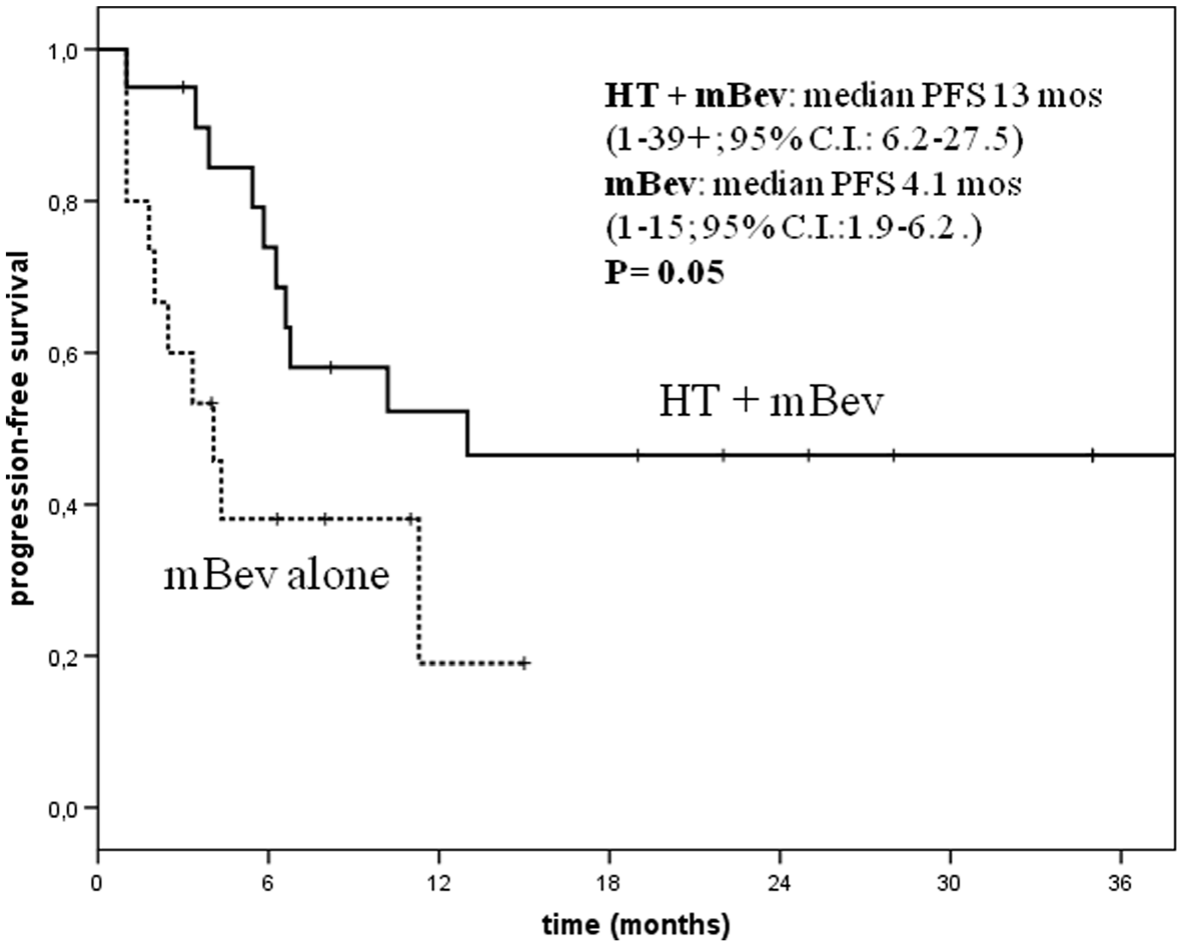
Progression free survival (PFS) according to maintenace Bevacizumb (mBev) combined or not to hormonal therapy (HT) in hormonal receptor positive patients.

Not all women who responded to BT and having tumors expressing hormonal receptors were administered HT as maintenance. The median mBev PFS of patients given HTBev was 13 months (1-39+; 95%C.I.: 6.2-27.5), while the median mBev PFS of patients who received only mBev was 4.1 months (1–15; 95%C.I.: 1.9-6.2). A statistically significant difference in terms of PFS was seen between these two groups of patients (p = 0.05) [Figure 1].

### Safety profile

The safety profile shown in all 35 patients during BT and mBev therapy can be considered acceptable (Tables 3 and 4). Overall, the incidence of severe grade of adverse events was higher during the maintenance therapy. The most common effects reported with mBev treatment were grade 2 (8.5%, in 2 HTBev and 1 bevacizumab alone patients) and grade 3 proteinuria (11.4%, 1 HTBev and 3 bevacizumab alone patients); grade 2 (5.7%, 1 HTBev and 1 bevacizumab alone patients) and grade 3 hypertension (8.5%, in 2 HTB and 1 bevacizumab alone women). Only 1 patient, affected by grade 1 hypertension before entering the study, experienced a worsening of the grade 3 hypertension. One case (bevacizumab alone) of pneumonia was reported in course of mBev therapy.

**Table 3:** **Safety of Bevacizumab plus Paclitaxel (total patients *n*** **= 35)**

**Toxicity**	**Grade 1**	**Grade 2**	**Grade 3**	**Grade 4**
	***n*(%)**	***n*(%)**	***n*(%)**	***n*(%)**
Bleeding	1 (2.8)	-	1 (2.8)	-
Protenuria	-	-	-	-
Hypertension	-	2 (5.7)		-
Neurotoxicity	1 (2.8)	2 (5.7)	-	-
LVS disfunction	1 (2.8)	-	-	-

**Table 4:** **Safety of maintenance-Bevacizumab (total patients *n*** **= 35)**

**Toxicity**	**Grade 1**	**Grade 2**	**Grade 3**	**Grade 4**
	***n*(%)**	***n*(%)**	***n*(%)**	***n*(%)**
Bleeding	2 (5.7)		-	-
Protenuria	-	3 (8.5)	4 (11.4)	-
Hypertension	1 (2.8)	2 (5.7)	3 (8.5)	-
Neurotoxicity	1 (2.8)	-	-	-
LVS disfunction	1 (2.8)	-	-	-
Other events			Pneumonia 1 (2.8)	

During the mBev, 3 (8.5%) and 4 (11.4%) patients discontinued treatment due to grade 3 hypertension and proteinuria at a median of 10 cycles (4–28). No patient resumed bevacizumab as maintenance treatment. All patients, but one, recovered from toxicity.No additional toxicity was observed in course of HTBev.

## Discussion

The peculiarity of this report is that provides further clinical results concerning the role of mBev in MBC women experiencing a clinical benefit by first-line BT combination. Moreover, no clinical finding has been reported yet on maintenance bevacizumab associated with hormonal therapy.

In the preclinical scenario, estrogen may play a key role in the regulation of angiogenesis. A direct effect of endocrine therapy on tumor vasculature has been reported. In a male mouse model of androgen-dependent breast cancer, castration led to tumor shrinkage and vascular regression [16]. However, endocrine resistance in this tumor model was suggested by a wave of neo-vascularization and tumor re-growth. On the other hand, endocrine castration initially caused a reduction in VEGF mRNA levels while, at the time of tumor re-growth, VEGF mRNA levels simultaneously rebound. Moreover, data coming from retrospective studies in patients with breast cancer indicated that high VEGF levels in breast tumor tissue were associated with decreased responsiveness to hormonal therapy in both adjuvant and metastatic breast cancer patients [20,21]. Overall, these data suggested that anti-VEGF therapy may delay or prevent the onset of endocrine therapy resistance in patients with hormone-sensitive breast cancer, supporting the use of the combined therapy with anti-angiogenic plus anti-hormonal agents in breast cancer. In our series, among patients with tumors expressing hormonal receptors, a better mBev PFS was seen when they were given an aromatase inhibitor plus bevacizumab compared with those who did not received any hormonal agent (mBev PFS 13 months vs 4.1 months, p = 0.05),

To date, few data exist regarding the efficacy and safety of bevacizumab as maintenance treatment in mBC. Smith et al. [22] have recently reported a subgroup analyses in patients enrolled in the Athena study who continued single agent bevacizumab after stopping chemotherapy. They found a median TTP and OS of 18.4 months and 30.0 months, respectively. In the majority of these patients, maintenance bevacizumab was administered alone for the extended periods and no informations were given about use of bevacizumab plus HT in hormonal receptors positive patients. In our prospective analysis on HER2-negative breast cancer patients treated with mBev beyond controlled disease after first-line BT therapy, the ORr (CR + PR, 62.8%) resembled those reported in large randomized phase III trials [7-9]. We administered bevacizumab in mBC beyond the end of chemotherapy until disease progression or unacceptable toxicity. The maintenance therapy with bevacizumab was effective, reporting the ORr of 48.5%, the median mBev PFS of 6.8 months (95%CI 0.8-12.7) and the duration of clinical benefit of 17.1 months (95%CI 12.2-21.9).

According to data coming from a recent meta-analysis, it could also be of interest that prior treatment with adjuvant taxanes was predictive of better mBev PFS [23]. In our analysis, the median mBev PFS was 10.2 months among patients who received a taxane as adjuvant treatment compared with 6.6 months among those who did not (P = 0.57). These findings are strengthen by the use of bevacizumab plus paclitaxel as first-line treatment also in women previously administered adjuvant taxane-including treatments.

Bevacizumab-associated adverse events are well known and include arterial thrombosis, wound-healing complications, gastrointestinal GI perforation, bleeding, hypertension, and proteinuria. A recent meta-analysis [24] of randomized, controlled bevacizumab-containing trials reported all grade hypertension in 3%to 36%of patients and proteinuria in 21%to 63%of patients with nephrotic-range proteinuria up to 2%of patients. The degree of proteinuria was bevacizumab dose-dependent. The pathogenesis of bevacizumab-associated hypertension and renal toxicity is poorly understood; however, one clinical study has suggested an association between these two adverse events [25]. An interesting explanation of the higher rate of proteinuria is that the continued inhibition of local VEGF in glomerular podocytes, as occurs in the prolonged bevacizumab treatment, leads to renal thrombotic microangiopathy, which causes the final event of proteinuria [26]. In our study, no evidence of increased risk of developing severe toxicities with longer bevacizumab treatment was reported, except for hypertension and proteinuria. According to data reported by Smith et al. (22), the onset of grade 3 proteinuria was common in the maintenance period. Similar findings were observed in advanced ovarian cancer, in patients receiving bevacizumab for prolonged time, after treatment with carboplatin, paclitaxel and bevacizumab [27]. In our study all patients recovered proteinuria toxicity after 2 weeks of bevacizumab discontinuation and all patients continued treatment. Grade 3 hypertension was infrequent (8.5%) and in that cases bevacizumab was continued in association with antihypertensive therapy. Adding hormonal therapy to bevacizumab did not cause any further toxicity.

Our study, despite the small number of patients, is the first prospective report on the activity and feasibility of a maintenance combination of bevacizumab with hormonal therapy in bevacizumab-pretreated women. In absence of data comparing association of HTBev versus HT alone, our study could be the basis for a comparison trial. Unfortunately, some questions remain still unanswered: 1) What are the most useful biomarkers to predict the efficacy during bevacizumab treatment? 2) What is the optimal subset of patients could benefit by mBev? 3) Is there the optimal hormonal treatment to combine with bevacizumab?

In the Athena trial, some studies explored potential biomarkers for bevacizumab, but more investigations are warranted. In the meantime, further studies including large number of patients, evaluation of their characteristics and analysis of response to previous antiangiogenic agents are needed, in order to select women who could benefit by mBev. Another missing information is the role of bevacizumab beyond progression, an issue that hopefully will be addressed by the ongoing TANIA study [28].

## Conclusions

Our results confirm that the strategy of prolonging the use of bevacizumab until progression is tolerable and often associated with encouraging long-term clinical outcomes. Moreover, based on the results coming from our series, in postmenopausal patients with hormone receptor-positive HER2-negative mBC, treatment with bevacizumab plus hormone therapy is feasible and effective, the most common treatment-related adverse events remaining hypertension and proteinuria, a toxicity profile which resembles that exerted by bevacizumab alone. Our findings support the use of the combination of bevacizumab plus anti-hormonal agents after bevacizumab plus paclitaxel in this setting of patients. However, considering the limits in terms of our analysis further investigations are needed in order to conclusively clarify this issue.

## Competing interests

The authors declare that they have no competing interests.

## Authors' contributions

AF was responsible for the conception of the study. AF, MR, GF and GM were responsible for the assembly and analysis and interpretation of data. AF and GF drafted the manuscript. DG did the statistical analysis. AF, MR, GF, GM, CN, PP, FDV, GD, FC provided study patients. AV did the radiological assessment and interpretation of it. All authors revised the manuscript critically and gave their approval for it to be published in this final version.

## Pre-publication history

The pre-publication history for this paper can be accessed here:

http://www.biomedcentral.com/1471-2407/12/482/prepub
